# Gut Microbiota and Inflammation Modulation in a Rat Model for Ulcerative Colitis after the Intraperitoneal Administration of Apigenin, Luteolin, and Xanthohumol

**DOI:** 10.3390/ijms25063236

**Published:** 2024-03-12

**Authors:** Patricia Magadán-Corpas, Álvaro Pérez-Valero, Suhui Ye, Sandra Sordon, Ewa Huszcza, Jarosław Popłoński, Claudio J. Villar, Felipe Lombó

**Affiliations:** 1 Research Group BIONUC (Biotechnology of Nutraceuticals and Bioactive Compounds), Departamento de Biología Funcional, Área de Microbiología, Universidad de Oviedo, 33006 Oviedo, Spain; magadanpatricia@uniovi.es (P.M.-C.); apv.moratalla@gmail.com (Á.P.-V.); yesuhui@uniovi.es (S.Y.); cjvg@uniovi.es (C.J.V.); 2 IUOPA (Instituto Universitario de Oncología del Principado de Asturias), 33006 Oviedo, Spain; 3 ISPA (Instituto de Investigación Sanitaria del Principado de Asturias), 33006 Oviedo, Spain; 4 Department of Food Chemistry and Biocatalysis, Wrocław University of Environmental and Life Sciences, Norwida 25, 50-375 Wrocław, Poland; sandra.sordon@upwr.edu.pl (S.S.); ewa.huszcza@upwr.edu.pl (E.H.); jaroslaw.poplonski@upwr.edu.pl (J.P.)

**Keywords:** inflammatory bowel disease, gut microbiota, flavonoid, anti-inflammatory

## Abstract

Ulcerative colitis (UC) is a chronic inflammatory disorder affecting the colon, with symptomatology influenced by factors including environmental, genomic, microbial, and immunological interactions. Gut microbiota dysbiosis, characterized by bacterial population alterations, contributes to intestinal homeostasis disruption and aberrant immune system activation, thereby exacerbating the inflammatory state. This study assesses the therapeutic efficacy of intraperitoneal (IP) injected flavonoids (apigenin, luteolin, and xanthohumol) in the reduction of inflammatory parameters and the modulation of the gut microbiota in a murine model of ulcerative colitis. Flavonoids interact with gut microbiota by modulating their composition and serving as substrates for the fermentation into other anti-inflammatory bioactive compounds. Our results demonstrate the effectiveness of luteolin and xanthohumol treatment in enhancing the relative abundance of anti-inflammatory microorganisms, thereby attenuating pro-inflammatory species. Moreover, all three flavonoids exhibit efficacy in the reduction of pro-inflammatory cytokine levels, with luteolin strongly demonstrating utility in alleviating associated physical UC symptoms. This suggests that this molecule is a potential alternative or co-therapy to conventional pharmacological interventions, potentially mitigating their adverse effects. A limited impact on microbiota is observed with apigenin, and this is attributed to its solubility constraints via the chosen administration route, resulting in its accumulation in the mesentery.

## 1. Introduction

Inflammatory bowel disease (IBD) is a term that encompasses two conditions, Crohn’s disease (CD) and ulcerative colitis (UC), both characterized by chronic inflammation of the gastrointestinal tract. While CD can affect any part of the intestinal tract (from mouth to anus), UC only affects the colon, causing abdominal pain, mucus, diarrhea, and blood stool [1]. IBD represents a group of archetypal complex disorders distinguished by chronic and varied symptoms, influenced by the interplay among environmental, genomic, microbial, and immunological factors [2].

Multiple investigations have corroborated notable distinctions in the composition, diversity, and/or abundance of gut microbiota (dysbiosis) between healthy individuals and those with IBD, leading to the loss of intestinal homeostasis or improper immune activation [3,4,5]. A lot of research is being conducted to better understand how the composition of the gut microbiota may influence the development and progression of IBD and existing common microbial signatures shared among patients with this pathology, such as an increase in the phylum *Pseudomonadota* [6,7]. The gut microbiome dysbiosis present in IBD patients is closely related to inflammation as it can increase the expression of inflammatory cytokines by the intestinal T lymphocytes. Several mediators contribute to the development of this chronic intestinal inflammation, with the primary ones being the interleukins IL-1β, IL-6, and IL-17 [8,9,10,11]. Additionally, the disruption of the mucosal barrier associated with IBD leads to alterations in the taxa composition of mucus communities. Commensal microorganisms may transition into pathogenic entities (pathobionts), thereby initiating and perpetuating the inflammatory process through aberrant activation of the mucosal immune system [12,13,14]. Furthermore, the production of short-chain fatty acids (SCFAs), generated from the metabolism of certain microorganisms on the mucus layer and dietary prebiotic carbohydrates, is also compromised. These SCFAs are associated with anti-inflammatory properties, the maintenance of normal mucosal function, and the regulation of intestinal immune homeostasis [12,15,16,17]. It has not yet been possible to determine whether the dysbiosis associated with IBD is the cause or a consequence of this pathology. Nevertheless, it is certain that the deregulation of the gut microbiota balance contributes to the evolution and progression of the IBD, supporting and maintaining the inflammatory responses.

At the clinical level, UC requires long-term potent pharmacological treatment [18], and it may be difficult to find a suitable medication without serious side effects. Conversely, flavonoids constitute a family of natural polyphenolic compounds derived from plants, which have been previously demonstrated to have anti-inflammatory properties. In this work, we have evaluated the effectiveness of three intraperitoneal (IP) injected flavonoids (apigenin, luteolin, and xanthohumol) in the amelioration of the symptoms associated with a UC rat model after dextran sodium sulfate (DSS) induction, as well as their influence on the gut microbiota composition. These three flavonoids have already been proven as antitumor compounds in previous in vitro experiments with human colon cancer cell lines [19], and their effectiveness in the treatment of IBD after oral administration has been shown in vivo [1,20,21,22,23]. Meanwhile, their therapeutic potential through IP administration remains an area warranting further comprehensive investigation to encompass the direct effects of flavonoids. This approach circumvents the initial gastrointestinal (GI) transit, thereby avoiding processes of degradation and modification of these biopharmaceutical compounds [24]. Studies also reported IP administration of small molecule pharmacological agents with a faster and more complete absorption, compared to oral routes [25]. Furthermore, a diminished dosage of flavonoids is required.

In addition, the impact of xanthohumol on the gut microbiota remains unexplored. In this work, we have characterized, for the first time, the gut microbiota changes induced by xanthohumol in a UC murine model. 

The primary aim of this research was to assess the potential therapeutic efficacy of IP administration of flavonoids as a novel and potential therapeutic intervention for UC in a rat model of this disease. In pursuit of this goal, multiple parameters relevant to the progression of UC have been evaluated, including gut microbiota characterization. This approach has encompassed both the direct effects of flavonoids and their indirect effects mediated through the modulation of gut microbiota populations. The changes in the gut microbiota composition caused by the flavonoid treatments have been associated with specific bacterial taxa. These alterations in the bacterial populations may therefore modify the metabolic pathways of the production of bioactive metabolites [26,27]. Finally, these breakdown metabolites (e.g., SCFAs, aromatic flavonoids breakdown derivatives, etc.) may act at different levels in the colon mucosa, downregulating the inflammatory status (e.g., at the level of lymphocyte population modulation, myeloperoxidase, cytokines, barrier function, etc.)

The experiments described in this work demonstrate that luteolin, and to a lesser extent xanthohumol, are successful in the treatment of UC via IP injection. Our main findings are that both flavonoids exert their activity over the modulation of the gut microbiota community structure towards a decrease in pro-inflammatory taxa and an increase in anti-inflammatory taxa. Some of these anti-inflammatory taxa are well-known producers of beneficial compounds, such as SCFAs and flavonoid metabolites, which may further contribute to modulating pro-inflammatory cytokines in the intestinal mucosa. Statistically significant differences have been observed in the reduction of the cytokines IL-6 and IL-1β in the three flavonoid treatment cohorts, likely linked to either the direct action of flavonoids or the metabolites resulting from the gut microbiota modulation. Finally, the luteolin observed effects are further extended to an improvement in colon ulceration and stool consistency parameters. However, in this study, IP administration has caused a reduced bioavailability of apigenin (and reduced protection against UC) due to the presence of precipitate granules in the mesentery, a fact that had not been previously described, and is probably derived from its lower hydrophilicity (in comparison with luteolin).

## 2. Results

Comparisons of the analyzed parameters were conducted between the PBS (phosphate buffer saline) control cohort, serving as the disease model, and each of the cohorts receiving flavonoid treatments. The analyzed parameters encompassed daily body weight measurements, serving as an indicator of effective digestive function as animals with UC typically exhibit compromised nutrient absorption and consequent reductions in body weight. Additionally, the disease activity index (DAI), which integrates changes in body weight and stool consistency (including diarrhea severity and the presence of blood in feces) as clinical indicators of disease severity, was assessed. Following euthanasia at the conclusion of the experiment, three macroscopic histological parameters associated with inflammation were investigated: hyperplastic Peyer’s patches, which are lymphoid tissues in the small intestine that undergo macroscopic enlargement in response to inflammation; colon length, which typically diminishes during inflammatory conditions; and colon ulceration, indicative of mucosal alteration. Also linked to the inflammatory status, two types of cytokines (IL-1β and IL-6) were measured in plasma samples, and the myeloperoxidase concentration was evaluated in the colon mucosa (after tissue homogenization). Subsequently, the weight of the caecum was quantified, and bacterial populations within this organ were analyzed using 16S rRNA next-generation sequencing (NGS) on an Illumina platform.

### 2.1. Effect of Flavonoid Treatments on Body Weight and DAI

**Figure 1:**
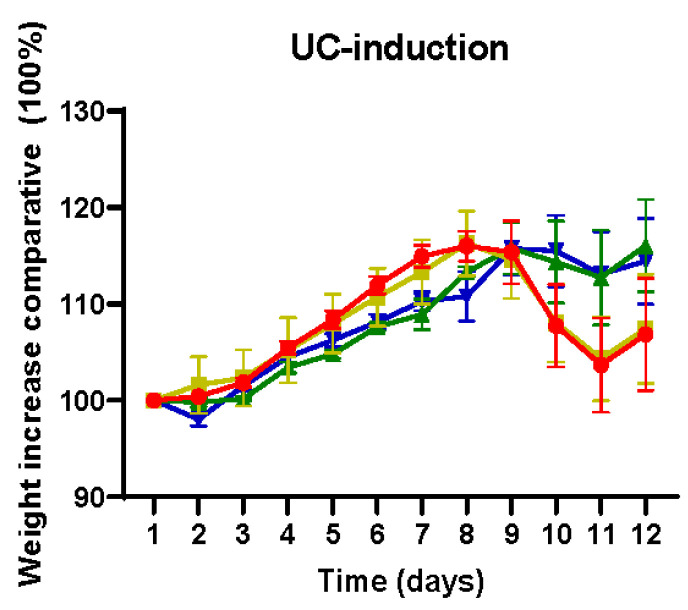
Comparison of the body weight increase in UC-induced animals from the four studied cohorts. The reductions in body weight observed around days 9 to 11 in UC-induced animals are due to the peak in UC symptoms (reduced feed ingest due to colon inflammation). Note that this reduction was minimal in luteolin and xanthohumol cohorts, thus showing a protective effect exerted by these treatments. PBS (red); apigenin (yellow); luteolin (green); xanthohumol (blue).

Body weight was monitored daily throughout the entire experimental period. Animals induced with UC exhibited discernible patterns in the comparison between cohorts (Figure 1). Notably, there was an initial increase in body weight until day 9, followed by a subsequent decline in all the cohorts, attributed to the induction of UC. It is noteworthy that this decline in body weight was particularly pronounced in the UC-induced animals from the non-treated (phosphate-buffered saline, PBS) and apigenin-treated cohorts, reaching its limit at day 11. All cohorts exhibited a recuperation phase toward the conclusion of the experiment as the effects of DSS treatment gradually waned.

By the end of the experiment (day 12), the mean body weight value for the UC-induced animals in the PBS cohort was 139.7 ± 15.9 g, while the two healthy absolute control animals reached 181.3 ± 20.1 g on average. Regarding the apigenin, luteolin, and xanthohumol cohorts, the mean weights at day 12 in the case of the UC-induced animals were 143.8 ± 19.4 g, 150.2 ± 8.3 g, and 149.6 ± 18.6 g, respectively, while their corresponding healthy controls weights were 183.4 ± 9.3 g, 170.6 ± 3 g, and 154.4 ± 17.1. Statistical analyses showed no significant differences between the PBS and either of the flavonoid treatment cohorts regarding body weight (Supplementary Figure S1a).

**Figure 2:**
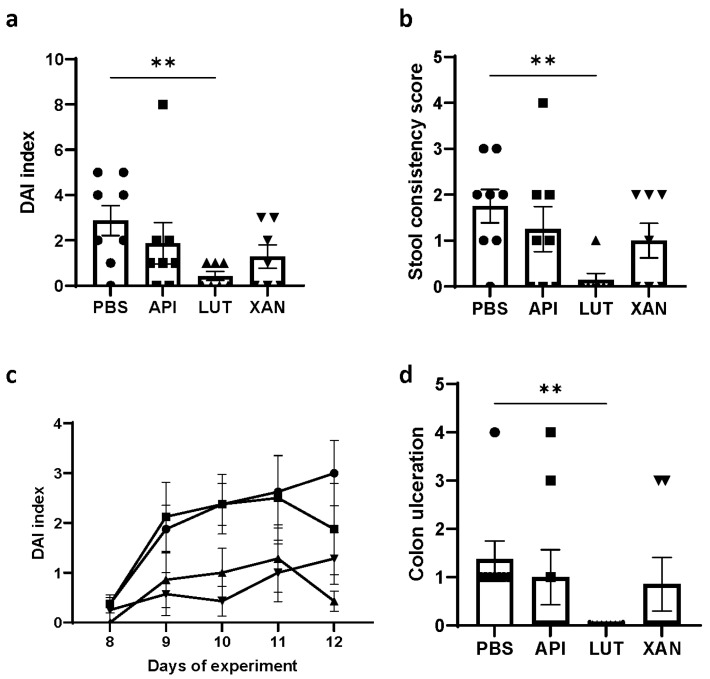
Measurements of several UC parameters in UC-induced animals from the four studied cohorts: (**a**) DAI index, (**b**) stool consistency score at day 12, (**c**) progression of the DAI index during the last five days of the experiment, and (**d**) colon ulceration at day 12. Comparisons were performed between the PBS control cohort and each flavonoid treatment cohort. In all cases, luteolin treatment showed a strong protective effect. Circle: control cohort; square: apigenin treatment cohort; upward triangle: luteolin treatment cohort; downward triangle: xanthohumol treatment cohort. Asterisks indicate statistically significant differences (** *p* < 0.005).

The mean DAI scores on day 12 are represented in Figure 2a, exclusively for the UC-induced animal groups. A statistically significant difference was observed when comparing the luteolin-treated cohort to the control PBS cohort. Given the absence of statistically significant variations in body weight across cohorts, the enhancement in the DAI index in the luteolin cohort primarily stemmed from an amelioration in stool consistency (Figure 2b), one of the contributing parameters for DAI computation.

Furthermore, DAI index trends over the final five days of this study (Figure 2c) revealed a reduction in DAI scores within the luteolin and xanthohumol treatment cohorts, consistently maintaining lower scores compared to those observed in the PBS and apigenin cohorts during the peak period of UC induction.

### 2.2. Effect of Flavonoid Treatments on Hyperplastic Peyer’s Patches, Colon Length, and Colon Ulceration

Peyer’s patches were macroscopically quantified along the small intestine after euthanasia as this lymphoid tissue becomes hyperplastic in response to inflammatory processes, showing rounded, protruding, white 2–3 mm ovals on the surface of the small intestine [28]. Their reduction in number could then be associated with lower inflammatory signals. A statistically significant lower number of hyperplastic Peyer’s patches was not observed in the flavonoid-treated animals compared to the PBS cohort regarding the UC-induced animals (Supplementary Figure S1b).

Colon length was also measured in all the surviving rats, as an indicator of colitis severity: the shorter the colon, the more severe the colitis inflammation. Again, no statistically significant differences were observed in the UC-induced animals between the PBS cohort and each of the three treatment cohorts (Supplementary Figure S1c). 

Finally, colon ulceration was quantified for each individual within this study, and the findings are represented in Figure 2d. A statistically significant disparity was observed in the reduction of colon ulceration between the group of animals that were induced with UC and received luteolin treatment and the untreated UC-induced animals from the PBS cohort. Notably, all animals within the luteolin cohort displayed a complete absence of ulceration.

### 2.3. Effect of Flavonoid Treatments on Pro-Inflammatory Cytokines and Myeloperoxidase (MPO)

**Figure 3:**
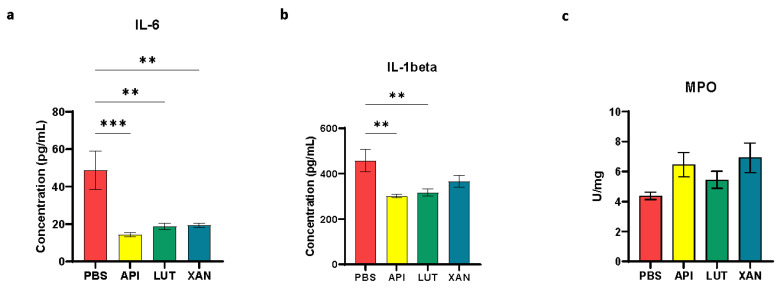
Measure of different pro-inflammatory biomarkers in UC-induced animals from the four studied cohorts: (**a**) IL-6, (**b**) IL-1β, and (**c**) MPO. Comparisons were performed between the PBS control cohort and each flavonoid treatment cohort. All three flavonoids demonstrated efficacy in reducing the levels of the pro-inflammatory cytokine IL-6, whereas only apigenin and luteolin exhibited effectiveness in modulating IL-1β. In contrast, flavonoids did not affect levels of MPO. PBS (red); apigenin (API, yellow); luteolin (LUT, green); xanthohumol (XAN, blue). Asterisks indicate statistically significant differences (** *p* < 0.005, *** *p* < 0.0005).

Statistical analyses revealed strong significant reductions in the pro-inflammatory cytokine IL-6 levels across all three flavonoid treatment groups compared to the PBS control cohort in the animals under UC induction. However, when examining cytokine IL-1β levels, xanthohumol did not exhibit statistically significant differences compared to the PBS control group, while apigenin and luteolin exhibited significant differences (Figure 3a,b). 

With respect to the enzymatic biomarker associated with colon mucosa inflammation (MPO), no statistically significant variances were detected in MPO tissue levels for either of the flavonoid treatments compared to the PBS control cohort (Figure 3c).

### 2.4. Caecum Weight and Metataxonomic Analyses of the Gut Microbiota

The caecum weight was assessed in the 38 surviving rats across the four cohorts, encompassing rats induced with UC. Statistical analysis revealed no significant differences in caecum weight in the comparison between the PBS cohort and each of the three treatment cohorts. (Supplementary Figure S1d).

In consideration of the microbiota studies, to assess the impact of flavonoid treatments on gut microbiota modulation subsequent to UC induction with DSS, comparisons were conducted on the composition of gut microbiota between the control PBS cohort and each specific flavonoid treatment cohort. Gut microbiota composition determination involved conducting a metataxonomics analysis of cecal content, utilizing 16S ribosomal RNA sequencing.

The two alpha diversity metrics, richness (observed OTUs: Operational Taxonomic Units) and evenness (a parameter that measures how numerically equal the bacterial community is in an experimental cohort, regarding the abundance and numbers of taxa), were measured within microbial communities, and alpha diversity was evaluated through the indices Chao1, Simpson, and Shannon. Boxplot representations of these indices are shown in Supplementary Figure S2. No statistically significant differences were found for any of these metrics between the UC-induced animals from the control PBS cohort and the flavonoid treatment cohorts, indicating no changes in terms of microbial alpha diversity.

**Figure 4:**
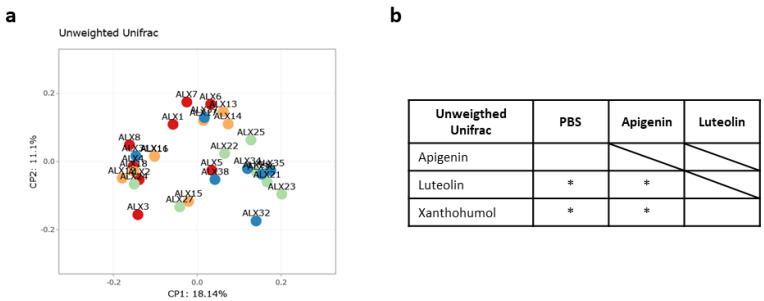
Comparisons of gut microbiota beta diversity metrics between the four studied cohorts: (**a**) unweighted Unifrac PCoA plot and (**b**) Permanova test for the unweighted Unifrac beta diversity measure. As ascertained by the unweighted Unifrac beta diversity analysis, the animals within the luteolin and xanthohumol cohorts exhibited greater similarity to each other, while they were more distinct from both the control (PBS) and apigenin cohorts. Conversely, the control and apigenin cohorts demonstrated similarity in terms of microbial community structure. In the PCoA plot, each dot represents one animal, and distances between dots represent the ecological distances between samples. PBS (red); apigenin (yellow); luteolin (green); xanthohumol (blue). Asterisks indicate statistically significant differences (* *p* < 0.05).

The unweighted Unifrac beta diversity index (a qualitative parameter that measures the structural composition of bacterial communities between experimental animal cohorts, including the taxonomy data) was also calculated to evaluate differences between groups in terms of species complexity. The principal coordinate analysis (PCoA) plot for the visualization of microbial communities’ structure is shown in Figure 4a. The analysis of beta diversity revealed notable statistically significant distinctions. Specifically, significant dissimilarities were discerned when contrasting the control PBS cohort with both the luteolin and xanthohumol cohorts (Figure 4b). Conversely, no statistically significant variations were evident when comparing the PBS cohort with the apigenin cohort. Furthermore, statistically significant disparities were also identified when comparing the apigenin cohort with both the luteolin and xanthohumol cohorts. However, there were no statistically significant distinctions between the luteolin and xanthohumol cohorts (Figure 4b).

**Figure 5:**
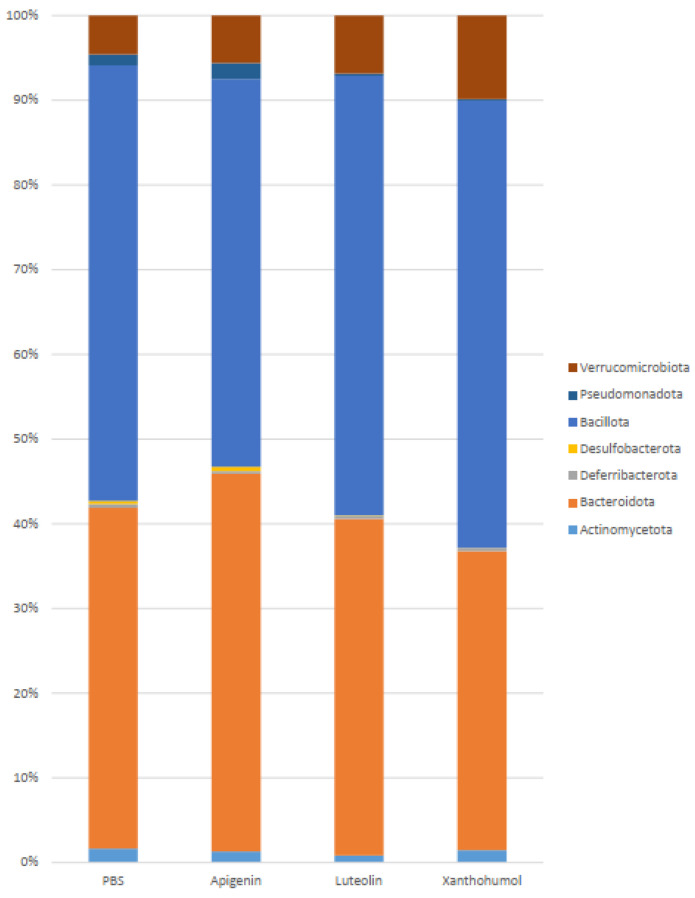
Barplot representation of the relative abundance (%) at the phylum level in the gut microbiota between animals induced with UC across the four experimental cohorts. *Bacillota* and *Bacteroidota* constitute 90% of the relative abundance in all cohorts. Higher differences between PBS and flavonoid treatment cohorts could be observed regarding the phyla *Pseudomonadota* and *Verrucomicrobiota*.

The metataxonomics analysis of the UC-induced animals’ gut microbiota showed statistically significant differences at different taxonomic levels and between different cohorts. In general, *Bacillota* and *Bacteroidota* constituted the most predominant phyla (90%) in all cohorts. The relative abundance of the other phyla varied depending on the treatment cohort (Figure 5, Table 1). 

When the UC-induced animals from the PBS cohort were compared to the UC-induced animals from each flavonoid treatment cohort (Figure 5, Table 1), statistically significant differences were observed, with a significant reduction in the phylum *Bacillota* in the apigenin cohort (45.49% vs. 51.20% in the PBS animals), while the phylum *Bacteroidota* was significantly decreased in the xanthohumol cohort (34.99% vs. 40.05% in the PBS cohort). A reduction in the phylum *Pseudomonadota* was observed in the luteolin (0.23%) and xanthohumol (0.17%) cohorts with respect to the control cohort (1.28%). The phylum *Desulfobacterota*, with the genus *Bilophila* as its unique member, was also significantly decreased in the xanthohumol cohort (0.03% vs. 0.36% in the PBS animals). Also remarkable, and statistically significant, was the increase in the phylum *Verrucomicrobiota*, with *Akkermansia muciniphila* as its unique representative species in the xanthohumol cohort (9.77% vs. 4.56% in the PBS cohort). 

Regarding the comparisons between the three flavonoid treatment cohorts, statistically significant differences were observed in the case of the phyla *Bacteroidota*, *Desulfobacterota*, *Bacillota*, and *Pseudomonadota* between the apigenin and the xanthohumol cohorts. Luteolin showed a statistically significant reduction in comparison with apigenin regarding phylum *Pseudomonadota*. 

**Figure 6:**
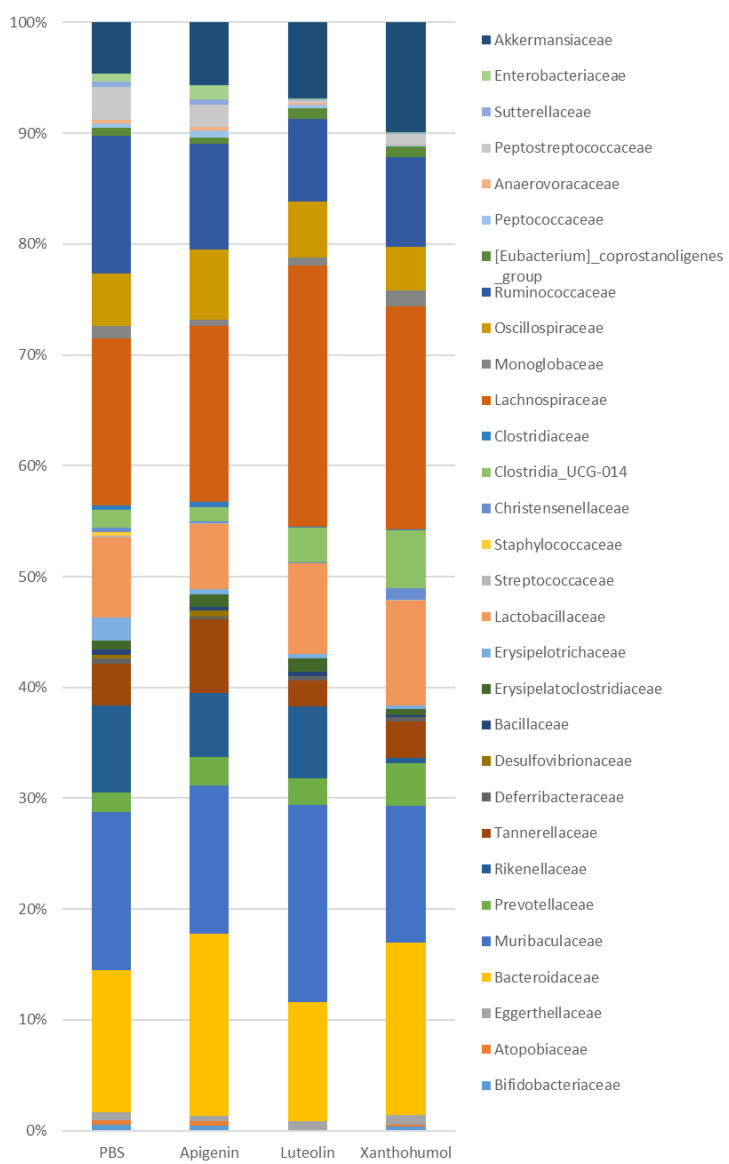
Barplot representation of the relative abundance (%) at the family level in the gut microbiota populations between the animals induced with UC across the four experimental cohorts. See Table 2 for a detailed list of statistically significant differences between the PBS control animals and each of the flavonoid treatments in the UC-induced animals.

The main differences at the family level in the comparison between the UC-induced animals from the PBS cohort and those treated with flavonoids were observed after luteolin and xanthohumol administration (Figure 6, Table 2, Supplementary Figure S3). The phylum *Bacillota* was significantly reduced only in the apigenin cohort. However, major changes were observed at the family level regarding the luteolin and xanthohumol cohorts. A statistically significant reduction was observed in the animals from all three flavonoid treatment cohorts regarding the families *Erysipelotrichaceae* (0.53% in the apigenin cohort, 0.38% in the luteolin cohort, and 0.36% in the xanthohumol cohort vs. 2.08% in the PBS cohort), *Streptococcaceae* (0.02%, 0.003%, and 0.02% vs. 0.16% in the PBS cohort) and *Staphylococcaceae* (genus *Staphylococcus*) (0.05%, 0, and 0.003% vs. 0.35% in the PBS cohort). The family *Peptococcaceae* showed a statistically significant reduction in the xanthohumol cohort (0.17% vs. 0.39% in the PBS cohort). Also statistically significant were the reductions in the luteolin and xanthohumol cohorts regarding the families *Anaerovoracaceae* (0.13% and 0.11%, respectively, vs. 0.33% in the PBS cohort), *Peptostreptococcaceae* (genus *Romboutsia*) (0.21% and 0.86% vs. 2.88% in the PBS cohort), and *Clostridiaceae* (genus *Clostridium sensu stricto 1*) (0.11% and 0.12% vs. 0.40% in the PBS cohort). Conversely, the family *Lachnospiraceae* was significantly increased in the luteolin cohort (23.25% vs. 14.96% in the PBS cohort). The decrease in the phylum *Bacteroidota* observed in the xanthohumol cohort could be mainly attributed to a reduction in the family *Rikenellaceae* (0.46% vs. 7.76% in the PBS cohort). A statistically significant increase was observed in the family *Prevotellaceae* in the xanthohumol cohort (3.83% vs. 1.73% in the PBS cohort). Two families from phylum *Pseudomonadota* were reduced in the UC animals from the luteolin and xanthohumol cohorts: *Sutterellaceae* (0.11% and 0.08%, respectively, vs. 0.54% in the PBS cohort) and *Enterobacteriaceae* (0.05% and 0.03%, respectively, vs. 0.68% in the PBS cohort) (Supplementary Figure S3). In the luteolin cohort, the family *Bifidobacteriaceae* (genus *Bifidobacterium*) (0.03% vs. 0.52% in the PBS cohort) showed a reduction (Supplementary Figure S3).

At the genus level (Table 3, Supplementary Figure S4), most of the differences were observed in the luteolin and xanthohumol cohorts compared to the PBS cohort. The genera *Turicibacter* and *Streptococcus*, as the most abundant ones of their respective families (*Erysipelotrichaceae* and *Streptococcaceae*), showed a statistically significant reduction in the luteolin and xanthohumol cohorts, while the genus *Clostridia UCG-014* was increased in the same cohorts (3.07% in luteolin cohort and 5.10% in xanthohumol cohort vs. 1.57% in the PBS cohort). The genus *Lachnospiraceae NK4A136* group (family *Lachnospiraceae*) showed a statistically significant increase in the luteolin cohort (15.48% vs. 6.19% in the PBS cohort), while the genus *Blautia*, belonging to the same family, was significantly increased in both the apigenin (0.30%) and the luteolin (0.37% vs. 0.07% in the PBS cohort) cohorts. The genus *Ruminococcus* (family *Ruminococcaceae*) showed a statistically significant reduction in the luteolin cohort (1.48% vs. 6.19% in the PBS cohort). The genus *Alistipes* (family *Rikenellaceae*) showed a high statistically significant decrease in the xanthohumol cohort (0.39% vs. 7.66% in the PBS cohort). The observed reduction in the phylum *Pseudomonadota* in the luteolin and xanthohumol cohorts was due to reductions in the genera *Parasutterella* and *Escherichia-Shigella*. The genus *Adlercreutzia* (family *Eggerthellaceae*) showed a statistically significant reduction in the luteolin cohort (0.17% vs. 0.4% in the PBS cohort). Conversely, the genus *Enterorhabdus* showed a high increase in the luteolin and xanthohumol cohorts (0.55% and 0.46% vs. 0.17% in the PBS cohort) (Table 3, Supplementary Figure S4).

At the species level (Table 3, Supplementary Figure S5), an uncharacterized bacterium from the genus *Blautia* showed a statistically significant increase in all three flavonoid treatment cohorts (0.28% in the apigenin cohort, 0.33% in the luteolin cohort and 0.15% in the xanthohumol cohort vs. 0.01% in the PBS cohort). The observed changes in the family *Bifidobacteriaceae* are mainly associated with the species *Bifidobacterium animalis*, which was highly reduced in the luteolin cohort. The two species *Bacteoides dorei* (1.57% in xanthohumol vs. 0.87% in PBS cohort) and *Bacteroides thetaiotaomicron* (2.32% in xanthohumol vs. 1.31% in PBS cohort) showed an increase in the xanthohumol cohort, and *B. dorei* also increased in the apigenin cohort (1.59%), but decreased in the luteolin cohort (0.36%).

Regarding the comparisons between the three flavonoid treatment cohorts, the luteolin and xanthohumol cohorts only showed differences in the genera *Alistipes*, *Peptococcus*, and *Romboutsia* (Supplementary Figures S3 and S4). The apigenin cohort shared the same differences as the PBS cohort in comparison to the other two cohorts: *Bifidobacteriaceae*, *Atopobiaceae*, *Rickenellaceae*, *Desulfovibrionaceae*, *Staphylococcaceae*, *Clostridia UCG-014*, *Clostridiaceae*, *Peptococcaceae*, *Peptostreptococcacee*, *Suterellaceae*, and *Enterobacteriaceae*. The apigenin cohort showed main differences compared to the luteolin cohort regarding the genera *Parabacteroides* and *Christenellaceae R7* group and compared to the xanthohumol cohort in the genus *Christenellaceae R7* group.

## 3. Discussion

UC is characterized by chronic inflammation and ulcers in the colon mucosa. This study evaluated the anti-inflammatory potential of IP injection of three different flavonoids (apigenin, luteolin, and xanthohumol) in the treatment of UC, identifying any direct effect, as well as potential changes in the gut microbiota composition that may explain or correlate with any beneficial effect in the UC pathophysiology observed (cytokines levels, mucosa alterations, etc.). These three compounds have previously demonstrated in vitro antitumor activity against HT-29, HCT116, and T84 human colon cancer cell lines [19]. Furthermore, oral administration of these flavonoids to murine models showed in vivo efficacy in the treatment of IBD. Assessments included a reduction in anti-inflammatory markers, mitigation of colon injuries, restoration of intestinal barrier integrity, and downregulation of immune pathways. An evaluation of the effects on gut microbiota modulation was conducted solely for apigenin and luteolin [1,20,21,22,23]. Conversely, oral administration of quercetin failed in the co-treatment enhancement of the anticancer effect compared to IP administration, further emphasizing the potential advantageous features of IP injection. Additionally, increased levels of quercetin were observed in tumor tissues following IP administration [29]. Using the IP administration route in this work, these nutraceuticals avoid GI first-pass and they are first transferred to the mesentery capillaries; from there, they reach the portal vein towards the liver. From the liver, they are secreted via the bile duct to the small intestine, where they finally reach the gut microbiota, modulating the taxa present in the lumen and generating metabolism products (derived from the corresponding flavonoid skeleton), which may act at the colonocyte level or enter the portal circulation again [25].

In this study, a total of forty rats were utilized as a model for UC induced by DSS, and the progression of this condition was assessed across various parameters. The rats were divided into four cohorts, with three cohorts receiving IP injections of flavonoids and the fourth cohort serving as a control (receiving only PBS injections). Following UC induction, each flavonoid treatment was compared to the PBS control cohort in terms of efficacy. Biomarkers from body, tissue, and plasma samples were analyzed, alongside metataxonomic data of the gut microbiota. No adverse effects on the animals’ health were observed following flavonoid treatments, as indicated by biomarker comparisons between healthy control animals (those sentinels not subjected to UC induction) across the different cohorts.

Among the three evaluated flavonoids, the present study has shown a superior effectiveness of luteolin in mitigating UC across all measured parameters. In this regard, a total absence of colon mucosa ulceration (Figure 2d) and a high reduction in the DAI index (Figure 2a,c) could be assessed after luteolin administration, in accordance with previous observations by other authors using oral administration [1]. The reduction in the DAI index in the luteolin cohort could be mainly attributed to an improvement in the stool consistency score (Figure 2b) since no significant differences were observed in body weight gain between the different cohorts (Supplementary Figure S1a). Although not statistically significant, it was notable how the decrease in the body weight on days 9 to 11, due to the UC stage, was markedly more pronounced in the PBS and apigenin cohorts, indicating a more acute manifestation of UC in these two cohorts (Figure 1). 

The reductions observed in the pro-inflammatory IL-6 and IL-1β cytokines (Figure 3a,b) may be attributed to either the direct systemic anti-inflammatory properties of the flavonoids or the generation of bioactive metabolites resulting from the microbial metabolism of these flavonoids in the gut. Most known microbial gut metabolites derived from the flavones apigenin and luteolin comprise 3-(4′-hydroxyphenyl) propionic acid and 3-(3′,4′-dihydroxyphenyl) propionic acid, respectively, generated by the cleavage of C-ring, with phloroglucinol being released in both cases [24,26,27,30]. In the case of xanthohumol, the resulting gut metabolite is the potent phytoestrogen 8-prenylnaringenin [26]. These gut microbiota metabolites have been described as antioxidant, anticancer, and antimicrobial bioactives [31], as well as anti-inflammatory, and are able to inhibit the secretion of pro-inflammatory cytokines (TNF-α, IL-1β, and IL-6) [32]. In our particular assay, xanthohumol, which has been described as being degraded by some microorganisms present in the human gut microbiota [24,26], caused a reduction in IL-6 circulating levels but had no significant effect on the IL-1β plasma levels in the analyzed rats, while apigenin and luteolin, which are metabolized in a different way, seemed to have similar effects over both cytokines in our animal model for UC (Figure 3a,b).

According to the literature, the gut microbiota plays a critical role in the pathogenesis of UC [33,34,35]. Dysbiotic gut microbiota was reported to be necessary for inflammation, and thus, this is associated with the development and progression of this IBD [12,36]. In this regard, a metataxonomics analysis of the cecal content was performed and the gut microbiota composition was determined through 16S ribosomal RNA sequencing. As a result, some interesting changes in the gut microbiota composition have emerged here as the most significant ones among all the comparisons performed. Based on these findings, we can affirm that IP administration enables flavonoids to reach the colon lumen through mesenteric absorption into the portal vein, their subsequent transfer to the liver, and eventual secretion into the intestinal lumen via the bile duct, thereby exerting an effect on the intestinal luminal ecosystem.

Thus, while richness and evenness were similar in all the cohorts of this study (Supplementary Figure S2), interesting differences were observed in terms of beta diversity of gut microbiota composition between the different groups (Figure 4). These beta diversity analyses suggest that the PBS and apigenin cohorts showed a similar gut microbiota community structure, while the same cohorts are barely related to the luteolin and xanthohumol cohorts, which in turn, seemed to share a resemblance (Figure 4b). This observation also became apparent when a heatmap clustering of the samples based on the abundance of genera was depicted (Supplementary Figure S6). Subsequently, a comprehensive analysis of the microbiota reaffirmed this finding (Figure 6, Table 2 and Table 3).

Major statistically significant differences were observed in the comparison of UC animals’ gut microbiota after luteolin and/or xanthohumol administration compared to the control PBS cohort. These differences included reductions and increases in pro-inflammatory and anti-inflammatory bacteria, respectively. The genera *Turicibacter*, *Streptococcus*, *Staphylococcus*, *Clostridium sensu stricto 1*, *Romboutsia*, *Parasutterella*, and *Escherichia-Shigella* [37,38,39,40] were reduced both in the luteolin and the xanthohumol cohorts (Table 3, Supplementary Figure S4). The *Parasutterella* and *Escherichia-Shigella* genera (phylum *Pseudomonadota*) are considered part of an unstable gut microbial community, and they are associated with the genesis and development of IBD, as well as with chronic intestinal inflammation [37,41,42]. Conversely, the genera *Clostridia UCG-014* and *Enterorhabdus* [26,43] were increased in these luteolin and xanthohumol cohorts (Table 3, Supplementary Figure S4). In particular, the genus *Enterorhabdus* belongs to the polyphenol-degrading family *Eggerthellaceae* (phylum *Actinomycetota*), which is associated with health status and reported to potentiate the production of the previously mentioned bioactive phenolic metabolites [26,44,45].

Changes associated only with the xanthohumol cohort were also observed, including a reduction in the family *Peptococcaceae* and the genera *Alistipes* and *Bilophila* (Table 2 and Table 3) [28,46,47]. The genus *Alistipes*, highly reduced in the UC animals from the xanthohumol cohort, has been correlated with the development of dysbiosis and disease [48]. The genus *Bilophila* has been described to have a potential role in chronic inflammation [49], and it has been found to be reduced in animal models treated orally with anthocyanins [28]. In the phylum *Bacteroidota*, statistically significant increases were observed in the family *Prevotellaceae* in the xanthohumol cohort (Table 2, Supplementary Figure S3) [39,50], as well as in the species *Bacteroides dorei* and *Bacteroides thetaiotaomicron*, both being species reported with anti-inflammatory activity (Table 3, Supplementary Figure S5) [51,52]. The species *Akkermansia muciniphila* (phylum *Verrucomicrobiota*) is mainly considered a potential second-generation probiotic for the treatment of intestinal microbiome-associated diseases [53]. In this study, *A. muciniphila* was increased in the UC animals of the xanthohumol cohort (Table 3, Supplementary Figure S5). This species is a mucin-degrading bacteria reported to release monosaccharides, amino acids, and SCFAs into the environment, stimulating beneficial intestinal bacteria metabolic functions and contributing to the alleviation of microbial dysbiosis due to IBD [54,55,56].

Meanwhile, changes exclusively associated with the luteolin cohort included increases in the genera *Blautia* and *Lachnospiraceae NK4A136* group, both belonging to the family *Lachnospiraceae*, and reductions in the genus *Ruminococcus* (Table 3) [37,39,57,58]. The family *Lachnospiraceae* (Table 2) is one of the main butyrate-producing bacteria, and it has been associated with the control of gut inflammatory processes via the reduction of the expression of pro-inflammatory cytokines, the conversion of primary to secondary bile acids, and the resistance against the colonization of intestinal pathogens [59,60,61,62]. The increase in the genus *Lachnospiraceae NK4A136* group has been previously related to inflammation alleviation after polyphenol administration [63]. Interestingly, an uncharacterized bacterium was increased in all three flavonoid treatment cohorts (Table 3, Supplementary Figure S5). This species belongs to the genus *Blautia*, a commensal SCFAs-producing bacteria, with a role in maintaining the environmental balance in the intestine and in preventing inflammation by upregulating intestinal regulatory T_reg_ cells [57]. Although a significant reduction was observed in the common probiotic species *Bifidobacterium animalis* in the UC animals of the luteolin cohort in comparison with the PBS cohort ones, this was the result of a maintained abundance of this species in all animals (healthy controls and UC-induced) in the luteolin cohort (Table 3, Supplementary Figure S5). Consistent with this observation, the literature has previously reported a bacteriostatic effect of luteolin over lactic acid bacteria and *B. animalis* [64].

Collectively, although numerous studies have reported the effectiveness of apigenin in the amelioration of IBD [20,21], our investigations demonstrate that, in the context of this UC animal model, the outcomes associated with apigenin treatment closely resemble those of the control cohort administered with PBS. This similarity extends across multiple domains, including physiological parameters, tissue biomarkers, and microbiota analyses, but not in the case of cytokines, where apigenin administration actually causes significant statistical differences in plasma levels of IL-1β and IL-6 (a direct effect of flavonoids IP injections).

In contrast, luteolin, and to a lesser extent xanthohumol, emerged as promising therapeutic agents for the prevention and treatment of UC disease when administered via IP injection. Both luteolin and xanthohumol exhibited an ability to modulate the composition of the gut microbiota community, favoring the enrichment of anti-inflammatory taxa, capable of producing advantageous compounds, like SCFAs or phenolic metabolites derived from flavonoids, while concurrently reducing the presence of pro-inflammatory taxa within the intestinal lumen. They could potentially elicit their beneficial effects towards UC directly, and their effects can be mediated through the bioactive metabolites produced by the gut microbiota during flavonoid metabolism. 

A primary contributing factor for the observed lower efficacy of apigenin, with a profile more akin to the PBS cohort, compared to luteolin and xanthohumol, in terms of modulation of gut ecosystem, appeared to be its reduced bioavailability following IP administration. This assertion was substantiated by the conspicuous presence of apigenin precipitate granules dispersed throughout the mesentery after euthanasia (Supplementary Figure S7). These deposits of apigenin suggested that, in contrast to luteolin and xanthohumol, apigenin experienced limited translocation into the mesentery capillaries, thus preventing its ultimate entry into the gut lumen. This disparity in translocation could likely be attributed to a lower solubility of apigenin, stemming from its chemical structure, wherein it possesses only one hydroxyl group on ring C, in contrast to luteolin, which possesses two.

The primary limitation of this study was mainly related to the timing of euthanasia, which occurred several days after the peak of symptoms in the UC challenge, during the animals’ recovery period (day 12). This timing may have avoided the detection of peak concentrations of pro-inflammatory cytokines in plasma and MPO in colon mucosa. Conversely, the study of gut microbiota populations during the symptomatic peak of UC (days 7 to 8) would have rendered a substantially altered composition of the gut microbiota and structural changes in the colon mucosa. During this period, the potential therapeutic effects of the administered flavonoids may have been masked due to factors such as reduced food intake by the animals and significant alterations in the digestive tract caused by the presence of blood.

The main contributions of this work include the detection of a lower bioavailability in the case of apigenin when administered intraperitoneally, probably due to its lower hydrophilicity. Also, there was a strong reduction in IL-6 and IL-1β cytokines exerted by the flavonoids and a reduction in the UC-associated weight loss (a symptom caused by the DSS treatment during pathology onset) in the case of luteolin and xanthohumol. At the tissular level, the strong reduction caused by luteolin in the DAI index and colon ulceration is remarkable. Finally, numerous and very significant changes were exerted by luteolin and xanthohumol regarding colon microbiota modulation (a strong increase in some anti-inflammatory populations and a strong reduction in some pro-inflammatory and commensal taxa), an aspect that is entirely new for this pathology in the case of xanthohumol. 

## 4. Materials and Methods

### 4.1. Drugs and Chemicals

Dextran Sodium Sulfate (DSS, 40,000 g/mol) was purchased from VWR Chemicals (Madrid, Spain). Apigenin and luteolin were provided by Fluorochem (Dublin, Ireland). Xanthohumol was purified following a modified procedure described previously [65]. The same batch of spent hops, stored in high-density polyethylene (HDPE) industrial barrels and closed under a nitrogen atmosphere, was used. The modification involved only the initial extract preparation step as it was fully completed at the Department of Food Chemistry and Biocatalysis, Wrocław University of Environmental and Life Sciences laboratories. Eighteen kilograms of spent hops were extracted with 90 L of acetone in 0.2 kg:1.4 L batches, each made in a 2-L Erlenmeyer flask shaken for 3 h on a rotary shaker (120 rpm). The formed pulp was vacuum-filtered on Whatman filter paper no. 4 and concentrated using a laboratory rotary evaporator. The combined extracts were further subjected to polyphenol precipitation and Sephadex LH-20 column chromatography steps, resulting in 20.233 g of Xanthohumol (>98% purity by HPLC). The flavonoids were resuspended for injection in Tween 80 and phosphate-buffered saline (PBS) at a stock concentration of 5 mg/mL for apigenin and luteolin and 2.5 mg/mL for xanthohumol.

### 4.2. Animals

A total of 40 five-week-old male Fischer 344 rats (*Rattus norvegicus*), were maintained in the Animal Facilities at the University of Oviedo (authorized facility No. ES330440003591). All animal experiments were approved by the Ethics Committee of the Principality of Asturias (authorization code PROAE 29/2021).

The rats were provided by Charles River (Lyon, France). Sterile drinking water and standard pelleted feed (Teklad Irradiated Global 14% Protein Rodent Maintenance Diet) (Envigo, Gannat, France) were provided ad libitum. The animals were housed in a room under controlled temperature (21 °C) and humidity and 12 h light/darkness cycles.

### 4.3. Experimental Design

After being acclimated for 1 week, the animals were randomized into four cohorts of 10 animals each, according to the administered treatment. These animals were intraperitoneally injected with PBS (cohort 1, negative control) or the corresponding flavonoid treatment (10 mg/kg of body weight): apigenin (cohort 2), luteolin (cohort 3), or xanthohumol (cohort 4). The IP injections were administered daily since the start of the experiment and for a period of eleven days.

Eight rats from each experimental group underwent UC induction through the addition of 3% DSS to the autoclaved drinking water, which was administered ad libitum from the initiation of the experiment and for one week. The remaining two animals from each group served as absolute healthy control animals and were not subjected to UC induction. These absolute control animals were utilized as sentinels to assess the effects of the treatment compounds (flavonoids) on healthy animals and to detect any possible side (negative) effects. 

On day twelve, the animals were anesthetized (with isofluorane) (Zoetis, Madrid, Spain) and sacrificed (via pneumothorax). Two rats, designated as rat number 6 within the luteolin-treated cohort and rat number 3 within the xanthohumol-treated cohort, necessitated euthanasia on days eight and nine of the experiment, respectively, due to severe weight loss conditions.

The parameters that were monitored daily during the whole experiment included body weight, food and water intake, stool consistency, and rectal bleeding.

### 4.4. Tissue Sample Collection

After euthanasia, 2 mL of blood was extracted via heart puncture from each animal and centrifuged at 3000 rpm for 15 min and then, the plasmas were frozen. The small intestine was removed, and then, the hyperplastic Peyer’s patches were macroscopically quantified. The caecums were immediately weighed after extraction using a precision scale and then frozen at −20 °C. Finally, the colons were removed, opened longitudinally, and washed with PBS (VWR, Madrid, Spain) in order to macroscopically assess the length and ulceration status. Also, proximal and distant samples from colons were collected and frozen at −80 °C.

### 4.5. Histological Studies

The hyperplastic Peyer’s patches were counted along each small intestine. Their number in the experimental animals was compared with respect to the hyperplastic Peyer’s patches of the 2 absolute control animals from each cohort (animals 9 and 10).

The percentage of colon length reduction in the UC animals was compared with respect to the colon length of the 2 control (healthy) animals from each cohort.

Regarding the macroscopic score assessment of UC, this parameter was quantified as follows: 0—no symptoms; 1—local hyperemia but no ulceration; 2—ulceration without hyperemia; 3—ulceration and inflammation in only one site; 4—two or more ulceration and inflammation sites; 5—ulceration bigger than 2 cm; values 6 to 1—one score point per each 1 cm of extra ulceration [66].

### 4.6. Assessment of the Disease Activity Index (DAI)

In order to quantify the clinical evolution of the UC, the Disease Activity Index (DAI) [66] was used. This index is a numerical disease activity measurement comprising the sum of two parameters: changes in growth rate (0: more than 5% body weight gain; 1: less than 5% body weight gain and less than 5% body weight loss; 2: 5 to 10% body weight loss; 3: 10 to 20% body weight loss; 4: more than 20% body weight loss), and stool consistency score (0: normal feces; 1: loose stool; 2: watery diarrhea; 3: slimy diarrhea with little blood; 4: severe watery diarrhea with blood).

### 4.7. Pro-Inflammatory Cytokine Analysis in Plasma

IL-1β and IL-6 tests were performed in plasma samples, using commercial Rat IL-1β and IL-6 ELISA Kits (Diaclone, Besançon, France) and following the manufacturer’s instructions.

### 4.8. Myeloperoxidase (MPO) Assays

A 0.5-cm longitudinal section from each distal colon was excised, and this pro-inflammatory enzyme was quantified following a published protocol [66].

### 4.9. 16S rRNA Sequencing and Gut Microbiota Analysis

A metagenomics analysis of the stool samples, obtained from the caecums, was also performed. For this, the Pathogen Detection Protocol from the E.Z.N.A.^®^ Stool DNA Kit (VWR, Madrid, Spain) was used. Caecums were thawed on ice for 30 min, and then, 200 mg of feces from a middle section of each caecum were placed in a 25-mL tube to continue with the extraction protocol. Finally, 200 uL of genomic DNA were obtained and quantified using a BioPhotometer^®^ (Eppendorf, Madrid, Spain). The total DNA samples were frozen at −20 °C in order to be subsequently analyzed via the amplification and sequencing of the variable regions V3 and V4 of the 16S ribosomal RNA gene using Illumina MiSeq (Microomics Systems, Barcelona, Spain). Amplification was performed after 25 PCR cycles. A negative control of the DNA extraction as well as a positive Mock Community control were included to ensure quality control. These studies allowed us to describe and quantify microbial alpha and beta diversities, as well as the taxonomic profiles from phylum to species levels.

### 4.10. Microbiota Analysis

Phylotype data were used to calculate the alpha diversity metrics in order to analyze the diversity of microbial communities. Alpha diversity analysis was used to measure the community richness (observed Operating Taxonomic Units or OTUs), defined as the number of different phylotypes present in a community. Alpha diversity was also used to measure the community evenness, given as the Pielou’s evenness index, which quantifies how equal the community is numerically, taking into account the number and the abundance of phylotypes in a community. The Chao1 (species richness), Simpson (level of biodiversity), and Shannon (species diversity) indices were also calculated.

Phylotype and phylogenetic data were used to calculate the beta diversity metrics in order to assess the microbial community’s structure. A principal coordinate analysis (PCoA) was performed, based on unweighted Unifrac distance, a phylogenetic qualitative measure, in order to detect differences in beta diversity.

### 4.11. Statistical Methods

For the metataxonomics analysis, alpha diversity comparisons were performed using a linear model with the appropriate distribution (negative binomial model for Chao1, beta regression for Simpson, and linear model for Shannon). Beta diversity distance matrices were used to calculate PCoA and to make ordination plots using the R software package version 4.2.0. The significance of the groups present in the community structure was tested using the Permanova test. The differential relative abundance of taxa was tested using a linear model based on the negative binomial distribution and ANOVA. Biodiversity R version 2.14-1, PMCMRplus version 1.9.4, RVAideMemoire version 0.9-8, and Vegan version 2.5-6 packages were used for the different statistical analyses carried out. 

For the rest of the comparisons, outliers have been identified and excluded from the statistical analyses. The normality of the different variables was tested using Shapiro–Wilk’s test. In light of these results, the data were then expressed as the mean value ± standard error of the mean (S.E.M.), and parametric methods were used for statistical analyses. Differences between cohorts were tested by a one-way ANOVA (analysis of variance). When the quantitative data were not normal, the non-parametric Kruskal–Wallis test was used. The graphic representation was carried out using GraphPad Prism software (version 9, GraphPad Software, San Diego, CA, USA). In each case, a *p*-value < 0.05 was considered statistically significant (* *p* < 0.05; ** *p* < 0.005; *** *p* < 0.0005; **** *p* < 0.0001).

## Figures and Tables

**Table 1:** Average relative abundance (%) at the phylum level in the gut microbiota between animals induced with UC, across the four experimental cohorts. The statistically significant differences in the comparisons between the control PBS cohort and each flavonoid treatment cohort are depicted. API: apigenin; LUT: luteolin; XAN: xanthohumol. Asterisks indicate statistically significant differences (* *p* < 0.05; ** *p* < 0.005; *** *p* < 0.0005).

Phylum	PBS	Apigenin	Luteolin	Xanthohumol	PBS vs. API	PBS vs. LUT	PBS vs. XAN
*Actinomycetota*	1.65	1.31	0.83	1.45			
*Bacteroidota*	40.05	44.32	39.40	34.99			*
*Deferribacterota*	0.46	0.29	0.33	0.35			
*Desulfobacterota*	0.36	0.53	0.07	0.03			*
*Bacillota*	51.20	45.49	51.48	52.32	*		
*Pseudomonadota*	1.28	1.87	0.23	0.17		**	***
*Verrucomicrobiota*	4.56	5.57	6.80	9.77			*

**Table 2:** Average relative abundance (%) at the family level in the gut microbiota between animals induced with UC across the four experimental cohorts. Significant differences in the comparison between the control PBS cohort and each flavonoid treatment cohort are depicted. API: apigenin; LUT: luteolin; XAN: xanthohumol. Asterisks indicate statistically significant differences (* *p* < 0.05; ** *p* < 0.005; *** *p* < 0.0005; **** *p* < 0.0001).

Family	PBS	Apigenin	Luteolin	Xanthohumol	PBS vs. API	PBS vs. LUT	PBS vs. XAN
*Bifidobacteriaceae*	0.52	0.43	0.03	0.35		**	
*Atopobiaceae*	0.42	0.43	0.007	0.18		**	
*Eggerthellaceae*	0.68	0.43	0.79	0.89			
*Bacteroidaceae*	12.66	16.23	10.59	15.25			
*Muribaculaceae*	14.15	13.16	17.56	12.16			
*Prevotellaceae*	1.73	2.48	2.38	3.83			*
*Rikenellaceae*	7.76	5.71	6.39	0.46			****
*Tannerellaceae*	3.74	6.51	2.33	3.22			
*Deferribacteraceae*	0.46	0.29	0.33	0.35			
*Desulfovibrionaceae*	0.36	0.53	0.07	0.03			*
*Bacillaceae*	0.45	0.29	0.37	0.13			
*Erysipelatoclostridiaceae*	0.79	1.09	1.20	0.57			
*Erysipelotrichaceae*	2.08	0.53	0.38	0.36	*	**	**
*Lactobacillaceae*	7.08	5.77	8.04	9.31			
*Streptococcaceae*	0.16	0.02	0.003	0.02	*	***	*
*Staphylococcaceae*	0.35	0.05	0	0.003	**	***	****
*Christensenellaceae*	0.36	0.10	0.08	1.07	*	*	
*Clostridia UCG-014*	1.57	1.30	3.07	5.10		**	****
*Clostridiaceae*	0.4	0.43	0.11	0.12		***	**
*Lachnospiraceae*	14.96	15.65	23.25	19.75		**	
*Monoglobaceae*	1.08	0.59	0.71	1.41			
*Oscillospiraceae*	4.63	6.21	4.92	3.89			
*Ruminococcaceae*	12.28	9.39	7.38	7.93			
*Eubacterium*	0.75	0.58	0.93	0.97			
*Peptococcaceae*	0.39	0.63	0.35	0.17			**
*Anaerovoracaceae*	0.33	0.29	0.13	0.11		**	***
*Peptostreptococcaceae*	2.88	1.96	0.21	0.86		****	*
*Sutterellaceae*	0.54	0.45	0.11	0.08		**	****
*Enterobacteriaceae*	0.68	1.30	0.05	0.03		**	**
*Akkermansiaceae*	4.56	5.57	6.80	9.77			*

**Table 3:** Average relative abundance (%) at the genus and species levels in the gut microbiota between animals induced with UC across the four experimental cohorts. Significant differences in the comparison between the control PBS cohort and each flavonoid treatment cohort are depicted. Asterisks indicate statistically significant differences (* *p* < 0.05; ** *p* < 0.005; *** *p* < 0.0005; **** *p* < 0.0001). API: apigenin; LUT: luteolin; XAN: xanthohumol.

Genus Species	PBS	Apigenin	Luteolin	Xanthohumol	PBS vs. API	PBS vs. LUT	PBS vs. XAN
*Bifidobacterium*	0.52	0.43	0.03	0.35		**	
*B. animalis*	0.41	0.41	0.03	0.28		*	
*Adlercreutzia*	0.40	0.22	0.17	0.24		*	
*Enterorhabdus*	0.17	0.15	0.55	0.46		****	***
*Bacteroides*	12.66	16.23	10.59	15.25			
*B. dorei*	0.87	1.59	0.36	1.57	*	**	*
*B. thetaiotaomicron*	1.31	2.09	1.12	2.32			*
*Alistipes*	7.66	5.67	6.37	0.39			****
*Bilophila*	0.36	0.53	0.07	0.03			*
*Turicibacter*	0.85	0.35	0.10	0.22		****	*
*Streptococcus*	0.15	0.02	0.003	0.016		**	*
*Staphylococcus*	0.35	0.05	0	0.003	***	***	****
*Clostridia_UCG-014*	1.57	1.30	3.07	5.10		**	****
*Clostridium sensu stricto 1*	0.40	0.43	0.11	0.12		***	**
*Blautia*	0.07	0.30	0.37	0.19	*	*	
*Uncharacterized*	0.01	0.28	0.33	0.15	**	**	*
*Lachnospiraceae NK4A136*	6.19	7.32	15.48	12.27		*	
*Ruminococcus*	6.19	3.47	1.48	2.93		**	
*Peptococacceae uncultured*	0.36	0.53	0.31	0.17			***
*Romboutsia*	2.88	1.96	0.21	0.86		****	*
*Parasutterella*	0.54	0.45	0.11	0.08		**	****
*Escherichia-Shigella*	0.68	1.30	0.05	0.03		**	**
*Akkermansia*	4.56	5.57	6.80	9.77			*
*A. muciniphila*	4.56	5.57	6.80	9.77			*

## Data Availability

Publicly available datasets (metagenome sequences) were analyzed in this study. These data can be found in the NCBI SRA database with accession number PRJNA1068569.

## Supplementary Materials

The following supporting information can be downloaded at: https://www.mdpi.com/article/10.3390/ijms25063236/s1.
