# Supplementary material for: Gut Microbiota and Inflammation Modulation in a Rat Model for Ulcerative Colitis after the Intraperitoneal Administration of Apigenin, Luteolin, and Xanthohumol
Source: Int J Mol Sci. 2024 Mar 12;25(6):3236. doi: 10.3390/ijms25063236 (PMC10970206; doi:10.3390/ijms25063236)
Supplement: Supplementary file 1 [file ijms-25-03236-s001.zip › ijms-2864952 - Supplementary Material.pdf]

Supplementary Figures

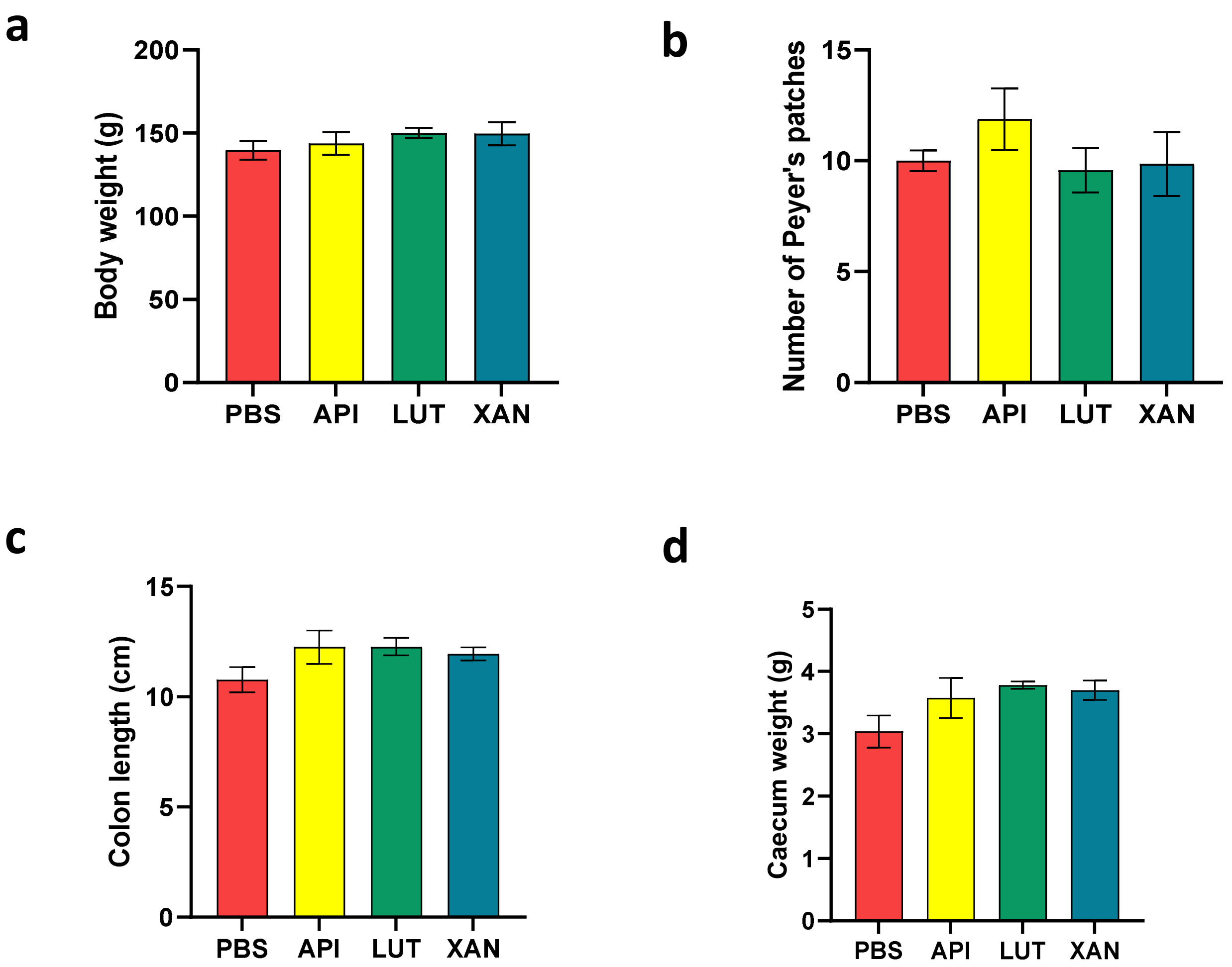

Figure S1. Measure of physical and inflammation parameters in UC-induced animals from the four studied cohorts: **(a)** mean body weight; **(b)** hyperplastic Peyer's patches; **(c)** colon length and **(d)** caecum weight between. Comparisons performed between PBS control cohort and each flavonoid treatment cohort. No statistically significant differences were observed in these comparisons. PBS (red); apigenin (API, yellow); luteolin (LUT, green) and xanthohumol (XAN, blue).

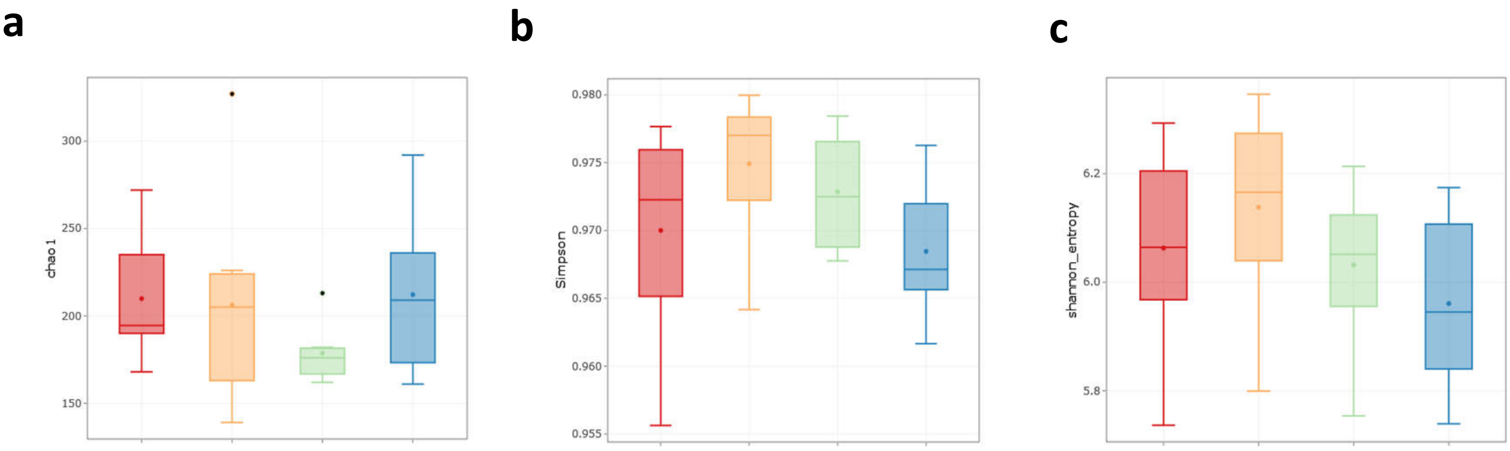

Figure S2. Boxplots of alpha diversity metrics **(a)** Chao1, **(b)** Simpson and **(c)** Shannon. The colored dot in each boxplot indicates the mean. Black dots indicate outliers. No statistically significant differences were found for any of these metrics among the UC-induced animals from the control PBS cohort and the flavonoid treatment cohorts, this indicating no changes in terms of microbial alpha diversity. PBS (red); apigenin (yellow); luteolin (green); xanthohumol (blue).

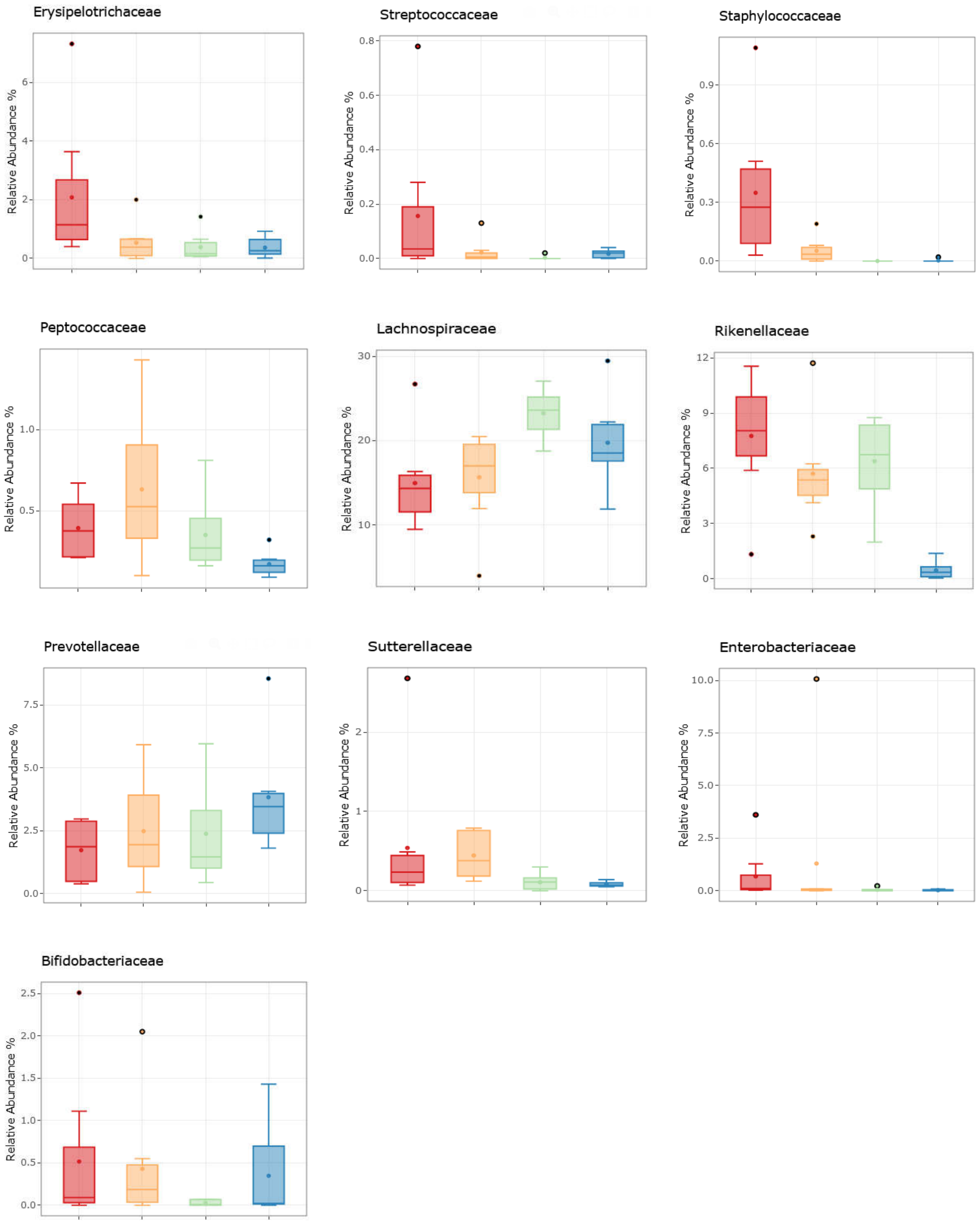

Figure S3. Boxplots of the relative abundance (%) of families with major significant differences regarding the comparison between UC-induced animals in the four studied cohorts. The colored dot in each boxplot indicates the mean. Black dots indicate outliers. PBS (red); apigenin (yellow); luteolin (green) and xanthohumol (blue).

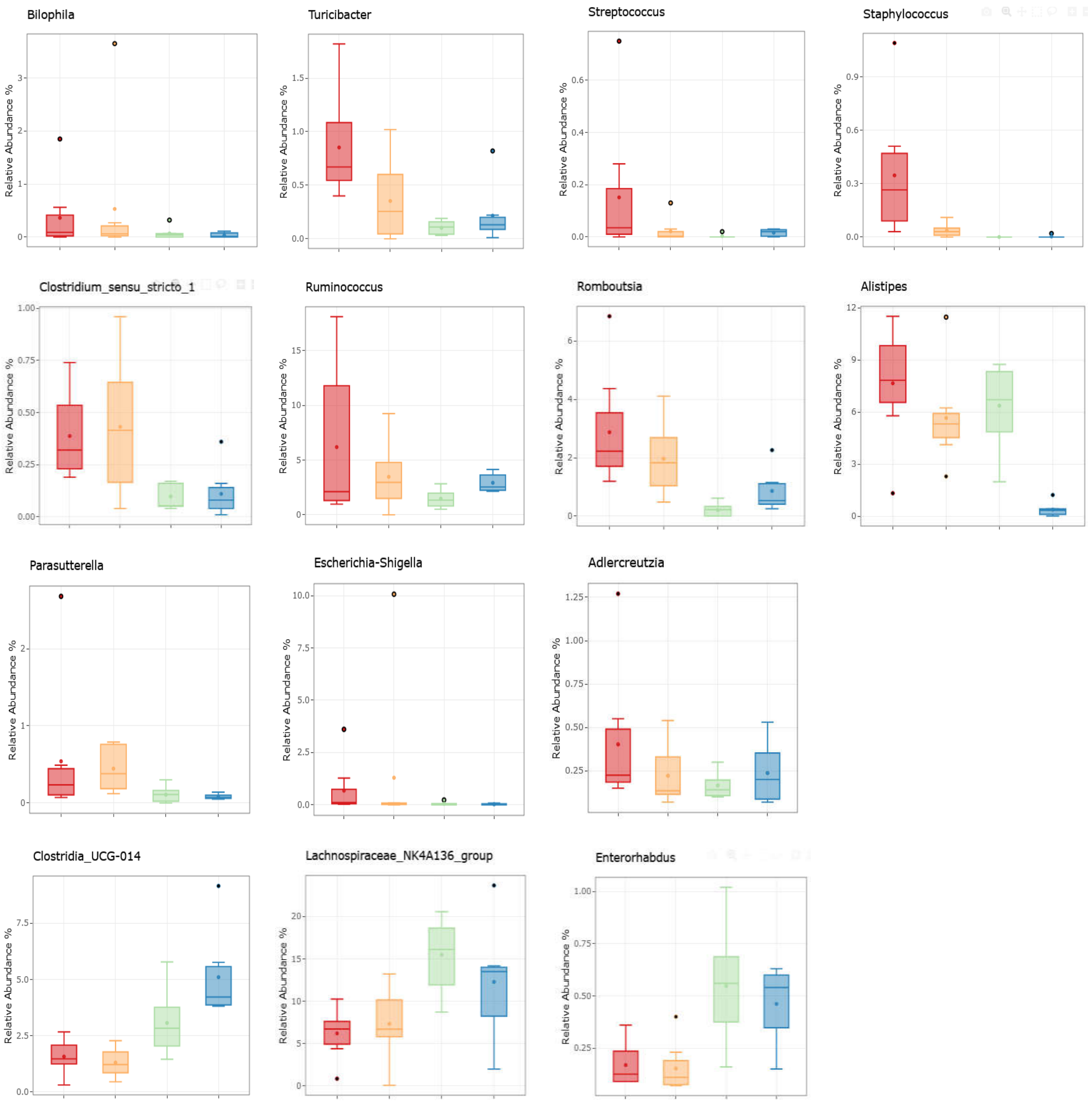

Figure S4. Boxplots of the relative abundance (%) of genera with major significant differences regarding the comparison between UC-induced animals in the four studied cohorts. The colored dot in each boxplot indicates the mean. Black dots indicate outliers. PBS (red); apigenin (yellow); luteolin (green) and xanthohumol (blue).

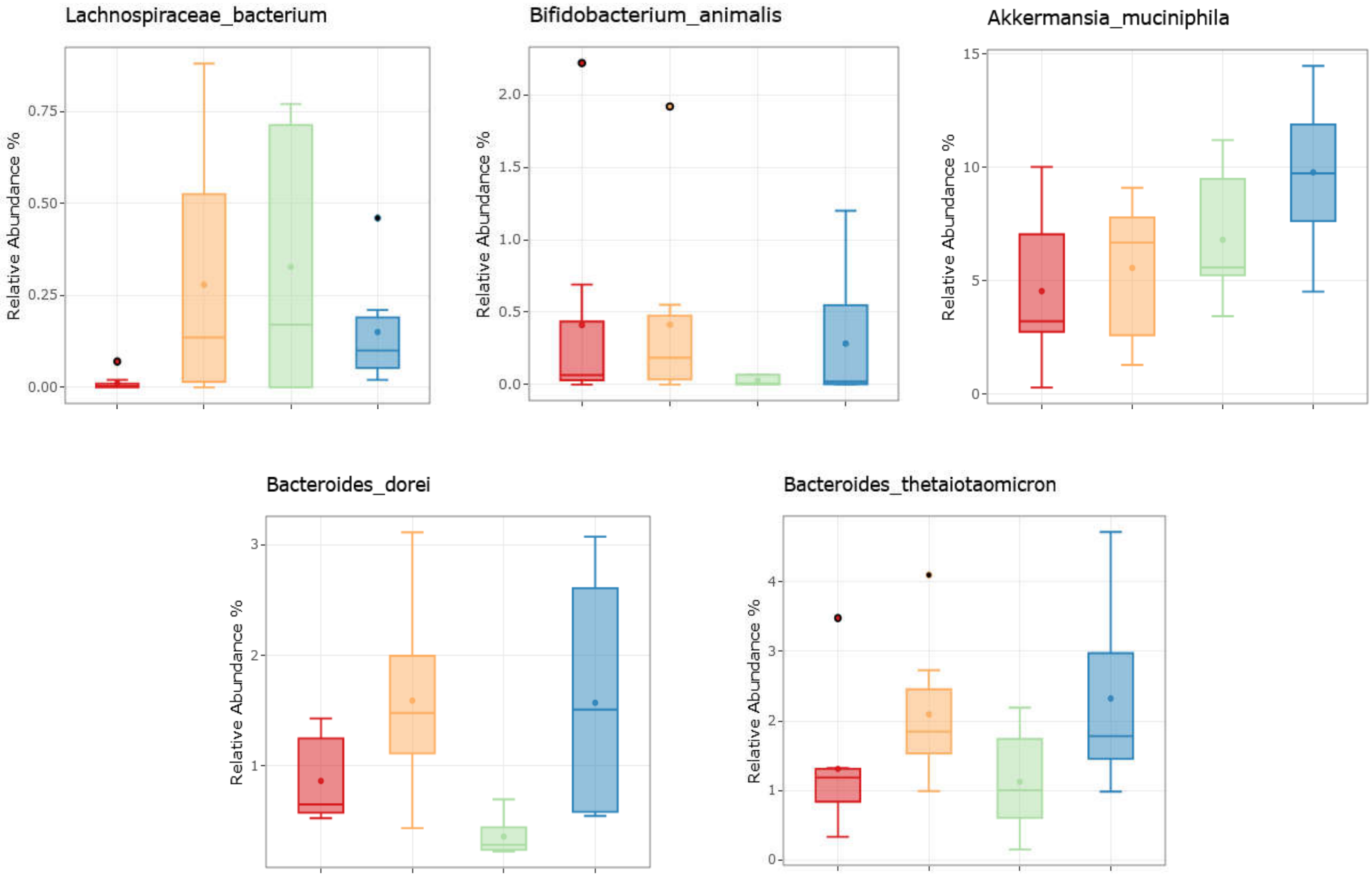

Figure S5. Boxplots of the relative abundance (%) of species with major significant differences regarding the comparison between UC-induced animals in the four studied cohorts. The colored dot in each boxplot indicates the mean. Black dots indicate outliers. PBS (red); apigenin (yellow); luteolin (green) and xanthohumol (blue).

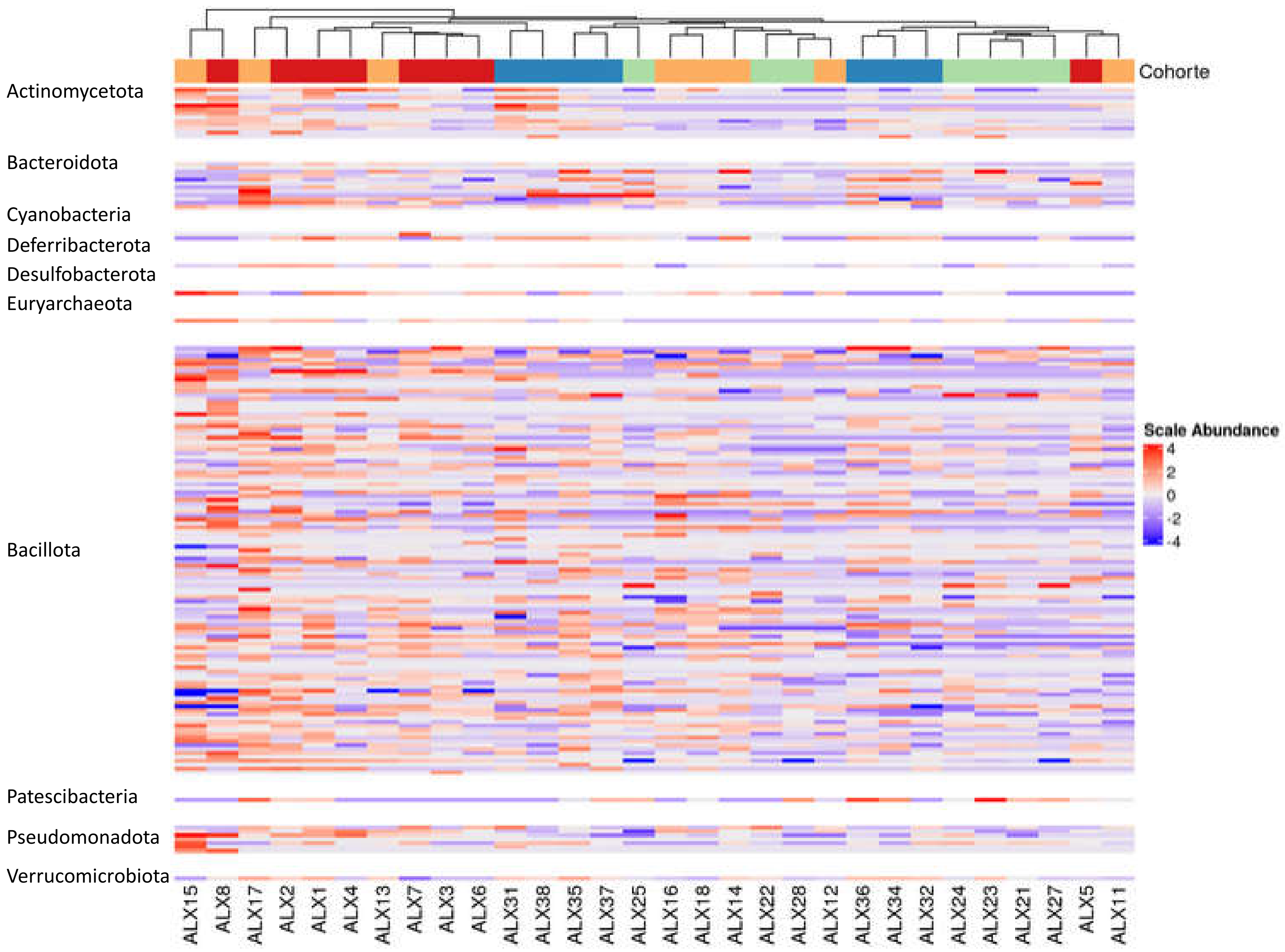

Figure S6. Heatmap representing the cohorts grouped based on the abundance of genera**.** Colors represent standardized abundances. Red means high abundance of the given genus, while blue means low abundance. Row labels add information of the phylum. PBS (red); apigenin (yellow); luteolin (green) and xanthohumol (blue).

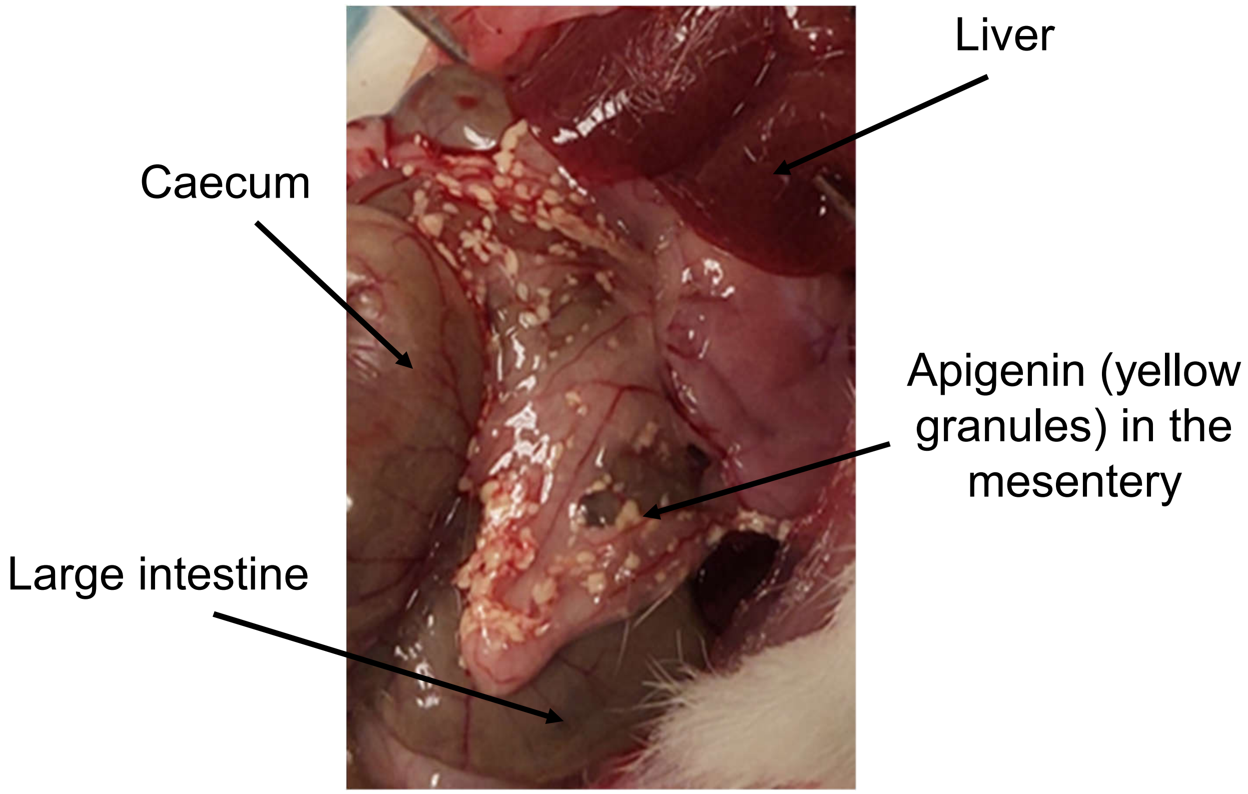

Figure S7. Apigenin precipitation granules in the mesentery of apigenin cohort rats.
